# A Diagnostics Platform for the Integrated Mapping, Monitoring, and Surveillance of Neglected Tropical Diseases: Rationale and Target Product Profiles

**DOI:** 10.1371/journal.pntd.0001746

**Published:** 2012-07-31

**Authors:** Anthony W. Solomon, Dirk Engels, Robin L. Bailey, Isobel M. Blake, Simon Brooker, Jia-Xu Chen, Jun-Hu Chen, Thomas S. Churcher, Chris J. Drakeley, Tansy Edwards, Alan Fenwick, Michael French, Albis Francesco Gabrielli, Nicholas C. Grassly, Emma M. Harding-Esch, Martin J. Holland, Artemis Koukounari, Patrick J. Lammie, Jacqueline Leslie, David C. Mabey, Mohamed Rhajaoui, W. Evan Secor, J. Russell Stothard, Hu Wei, A. Lee Willingham, Xiao-Nong Zhou, Rosanna W. Peeling

**Affiliations:** 1 London School of Hygiene & Tropical Medicine, London, United Kingdom; 2 World Health Organization, Geneva, Switzerland; 3 Imperial College London, London, United Kingdom; 4 National Institute for Parasitic Diseases, Shanghai, People's Republic of China; 5 Centers for Disease Control and Prevention, Atlanta, Georgia, United States of America; 6 Institut National D'Hygiène, Rabat, Morocco; 7 Liverpool School of Tropical Medicine, Liverpool, United Kingdom; Queensland Institute for Medical Research, Australia

## Background

Control and elimination strategies for trachoma, lymphatic filariasis, onchocerciasis, schistosomiasis, ascariasis, trichuriasis and hookworm infection have striking similarities, including the use of periodic mass drug administration (MDA). Because these diseases tend to be co-endemic in the poorest communities of the poorest countries, such that multiple NTDs are frequently found not just in the same populations but within the same individuals [1], it has been suggested that mapping, treatment, impact monitoring, and post-elimination surveillance could be coordinated to better utilise limited human and financial resources. Although many programmes now distribute multiple anthelmintics simultaneously, progress in integrating mapping [2], [3], [4], monitoring, and surveillance [5] activities has been slow [6]. Ideally, population sampling strategies, fieldwork protocols, and sample types (e.g., blood or urine) could all be harmonised between diseases to increase population compliance, simplify overall survey procedures, and decrease costs.

For each of these diseases, current diagnostic tools are imperfect (Table S1A), especially for areas with low prevalence. A cost-effective strategy for improved tool development would incorporate integration of diagnostic strategies from the outset [7], [8].

To review available methods for population-based assessment of NTDs, develop target product profiles for tools to monitor infection burden, and consider how those tools would be used in the context of disease elimination programmes, the London School of Hygiene & Tropical Medicine (LSHTM), in collaboration with the World Health Organization, held a consultation at LSHTM from July 19–20, 2010. Participants included disease experts, laboratory and field scientists, authorities on diagnostics, control programme managers, mathematical modellers, and health economists. By bringing together, for the first time, individuals with such a broad spectrum of intersecting disease- and discipline-specific interests to consider issues surrounding integration of diagnostic systems, the consultation aimed to improve on the usual vertical approach to tropical diseases research, encouraging formulation of an innovative approach.

This article summarises that consultation's outcomes, suggests target product profiles and a list of immediate research priorities, and drafts a road map for future efforts. We argue for development of a multiplex platform for NTD mapping, monitoring, and surveillance, and suggest changes to policy that might ensue if such a system were to become available.

## Evolution of Diagnostic Needs with Successful Programme Implementation

We conceptualise four time points or periods at which disease elimination programmes require diagnostics:

1. *Mapping* to establish baseline disease prevalence, facilitating targeting of interventions.
2. *Impact monitoring* after interventions have commenced.
3. The *stopping decision*, which determines whether the pre-defined elimination target has been reached, allowing discontinuation of interventions.
4. *Post-elimination surveillance* after intervention has ceased.

Mapping and impact monitoring may require both qualitative and quantitative data from each individual sampled, in order to generate information about the prevalence and intensity of infection. As the prevalence falls with successful control interventions, the intensity of infection also falls. Therefore, to detect the last remaining infections, a more sensitive test may be required. However, for some NTDs (e.g., trachoma), individuals with a low pathogen load are unlikely to transmit infection, so sensitivity is less important. Specificity becomes more important as disease prevalence decreases and is an absolute requirement in certifying elimination [5].

To enable meaningful interpretation of the effect of interventions, the sensitivity and specificity of diagnostic tools used in mapping should be about the same as the sensitivity and specificity of tools used in impact monitoring. One rational difference between mapping and impact monitoring may be the commissioning (in environments where high baseline infection prevalence is expected) of mapping surveys enrolling the same number of clusters over a larger area, in order to save resources. For example, province- or region-level baseline assessments of trachoma prevalence are now accepted for the purposes of requesting donated azithromycin in areas where active trachoma prevalence in 1–9-year-olds is expected (and then proven) to be higher than 10%; subsequent impact monitoring is performed at district level.

Ideally, stopping decisions would be based on documentation of the absence of transmission. In practice, however, these decisions are often made once there is documentation of absence of current or previous infection in a sentinel population, such as children born after the time transmission is believed to have been interrupted [9]. Stopping decisions require data generated using diagnostic tools whose specificity is at least as high as those used for mapping, to avoid unnecessarily prolonging MDA. Tool sensitivity is also crucial here to avoid premature MDA cessation and later rebound in infection prevalence. An assay considered adequate for mapping when prevalence is high may have inadequate sensitivity to detect infection in areas with low infection intensity.

Stopping decisions are made during the process of impact monitoring, and also mark the commencement of post-elimination surveillance. Data generated to inform stopping decisions should therefore provide useful comparators against both impact monitoring data and data that will subsequently be collected as part of surveillance activities.

In general, programmes are likely to require antigen- or nucleic acid–detection assays (to determine prevalence of current infection) for mapping and impact monitoring prior to elimination, and a combination of assays detecting antigens (or nucleic acid) and antibodies (to assess prevalence of exposure in particular population subsets) for post-elimination surveillance. For the worm infections, reliance on detection of transmission stages (eggs, microfilariae) becomes more problematic as the elimination endpoint is approached, since (other than for ascariasis) this will only identify hosts infected with both male and female adults. Specific detection of IgG subtypes may be useful in some cases, particularly if applied at population level: for example, IgG4 responses are characteristic of chronic helminth infections, and titres decline following successful therapy in lymphatic filariasis, onchocerciasis, schistosomiasis, and strongyloidiasis. Monitoring vector, intermediate host, or non-human reservoir [10] populations for the presence of parasites may be important in confirming elimination of infection.

Apart from performance characteristics, it is important to consider the operational characteristics of an assay. Large population-based surveys may require tests that can be batched for high throughput. Point of care tests, which generally detect antigen or antibody in dipstick or card format, are relatively cheap, require little formal operator training, and can be performed in the community [11]. They are of particular use when programme personnel need to make immediate decisions as to whether intervention is required. This is helpful when individual patients are being assessed. However, for MDA, where decisions are needed on whether or not to treat whole communities or districts, laboratory-based tests are probably adequate, provided samples are easy to collect (e.g., fingerprick) and transport (e.g., dried blood spots).

## Target Product Profiles and Immediate Research Priorities

Target product profiles for lymphatic filariasis, trachoma, schistosomiasis, onchocerciasis, and soil-transmitted helminths are shown for the mapping and impact monitoring phases in Table 1, and for the post-elimination surveillance phase (first four diseases only) in Table 2. The tables consider only the needs for diagnostic tools in mapping, monitoring, and surveillance of human infection because we see these as priorities for any first-generation integrated platform for NTD diagnostics; we have, for the moment, put aside programme requirements for monitoring MDA coverage; measures of morbidity; possible emergence of drug resistance; prevalence of infection in vectors, intermediate hosts, or reservoir animals; and force of transmission through environmental sampling.

**Table 1:** Proposed target product profiles for diagnostic tools for selected NTDs, mapping, and impact monitoring.

Characteristic	Lymphatic Filariasis	Trachoma	Schistosomiasis	Onchocerciasis	Soil-Transmitted Helminths
Intended use	Mapping, monitoring, and stopping decision	Mapping, monitoring, and stopping decision	Mapping, monitoring, and stopping decision	Mapping, monitoring, and stopping decision	Mapping and monitoring
Possible target population^a^	6–15-year-old children	1–9-year-old children (could be adjusted)	6–15-year-old children plus occupational groups	6–15-year-old children	6–15-year-old children
Possible sample types	Blood spot	Eye swab (other: mouth or nose swab, tears)	Blood spot or urine (avoid stool if possible)	Blood spot	Blood spot or urine (avoid stool if possible)
Ideal diagnostic marker	Parasite antigen	*C. trachomatis* antigen	Species-specific antigen OR pan-genus antigen	Parasite antigen	Parasite antigen
Ideal test format	POC or high throughput laboratory assay	POC or high throughput laboratory assay	POC assay	POC or high throughput laboratory assay	POC assay
Availability of ideal diagnostic marker	Available but not right format, low reliability, high cost, and temperature sensitive	Available but not right format	Not yet available	Not yet available. IgG4 antibody may be a reasonable proxy	Not yet available
Required performance characteristics	95% sensitive; *W. bancrofti*-specific	>50% sensitive, 99.5% specific	>50% sensitive, 99.5% specific	>50% sensitive, 99.5% specific	>50% sensitive, 99.5% specific
Comparator assay (current reference standard)	Night blood micro-filaraemia	Quantitative PCR	Kato-Katz (multiple slides and multiple days) and/or urine filtration	Skin snips to detect micro-filariae	Kato-Katz (multiple slides and multiple days)
Possible sampling strategies	PBPS/LQAS, school based, sentinel sites	PBPS/LQAS, home based, sentinel sites	PBPS/LQAS, school based, 50/school, increasing with control	PBPS/LQAS	PBPS/LQAS, school based

LQAS, lot quality assurance sampling; NTDs, neglected tropical diseases; PBPS, population-based prevalence survey; PCR, polymerase chain reaction; POC, point of care.

a Based on peak infection prevalence, convenience, or both.

**Table 2:** Proposed target product profiles for diagnostic tools for selected NTDs, post-elimination surveillance.^a^

Characteristic	Lymphatic Filariasis	Trachoma	Schistosomiasis	Onchocerciasis
Intended use	Post-elimination incidence of infection	Post-elimination incidence of infection	Post-elimination incidence of infection	Post-elimination incidence of infection
Possible target population	Children born after transmission interruption	Children born after transmission interruption	Children born after transmission interruption	Children born after transmission interruption
Possible sample types	Blood spot	Blood spot	Blood spot or urine (avoid stool if possible)	Blood spot
Ideal diagnostic marker	Antibody	Antibody to a conserved species-specific epitope of MOMP	Antibody	Ov16 antibody
Availability of ideal diagnostic marker	Not available	Libraries available	In development	Available, but additional validation needed
Ideal test format	High throughput laboratory assay	High throughput laboratory assay	High throughput laboratory assay	High throughput laboratory assay
Population infection thresholds (for stopping MDA)	1%	Not defined	10% of school-aged children	1/3,000
Probable sampling strategy	PBPS	PBPS	PBPS or school surveys (or sentinel occupations)	PBPS

a Schistosomiasis is included in this table because several countries have programmes to eliminate this disease [18], [19]. The soil-transmitted helminth infections are not included because (as for schistosomiasis in most endemic states) the current goal is prevention of morbidity in school-aged children through periodic high-coverage MDA.

ICT, immunochromatographic card test; LF, lymphatic filariasis; MDA, mass drug administration; MOMP, major outer membrane protein of *C. trachomatis*; NTDs, neglected tropical diseases; PBPS, population-based prevalence survey.

The target product profiles that we set out here are aspirational. Some tests (e.g., antigen assay for *W. bancrofti* [Table 1] or Ov16 antibody assay in previously onchocerciasis-endemic areas [Table 2]) appear close to being validated for programme use, while for others, numerous technical hurdles remain. For this reason, we expect some of our target product profiles—particularly blood- or urine-based antigen detection tests for the soil-transmitted helminthiases—to be controversial. However, there is presently at least one commercially available ELISA kit to detect IgG directed against *Ascaris lumbricoides* in human serum: it should be possible to develop a test to detect the antigen driving that antibody response. If such antigens only circulate briefly in the early part of the *Ascaris* life cycle, this may actually be helpful in interpreting test results at community level, since antigen detection will indicate the presence of ongoing transmission. Immediate research priorities are shown in Table 3.

**Table 3:** Immediate research priorities.

Disease	Research Goal	Feasibility (0–10^a^: 0, Impossible; 10, Inevitable)	Impact if Achieved (0–10^a^: 0, None; 10, Massive)
Lymphatic filariasis	Development of antigen tests to usable/reliable format	9	8 if ≤USD 0.50
	Development and validation of tests (e.g., IgG4-subclass antibody detection tests using recombinant Bm14, BmR1, WbSXP, and *W. bancrofti*-specific antigens [20] or PCR-based detection of parasite DNA in homogenised mosquitoes [21]) useful for post-elimination surveillance, with accompanying standardised survey methodologies	9	8 if ≤USD 0.50
Trachoma	Development of a test for ocular *C. trachomatis* infection [22] able to maintain specificity at high temperatures and low humidity [23]	9	8 if ≤USD 0.50
	Development of eye/nose swab-, saliva-, or blood-based anti-*C. trachomatis* antibody test and exploration of the impact of successful trachoma control on antibody profiles in endemic populations	3	5
	Development and validation of a school-based survey protocol (need threshold minimum school attendance)	7	8
Schistosomiasis	Development of antigen [24] or antibody [25] isotype combination(s) useful in high and low transmission intensity environments, able to distinguish current from past infection	8	9
	Development of antigen or antibody isotype combination(s) to distinguish between different species	8	4
	Development of serum markers of morbidity	6	8
Soil-transmitted helminthiases	Development of reliable blood- or urine-based assays for detection of current infection	4	9
	Development of serum markers of morbidity	6	8
Onchocerciasis	Development of a quantitative antigen test for use in endemic areas in Africa and validation of Ov16 antibody test for demonstrating interruption of transmission in Africa	5	8
	Development of a test for loaiasis	5	9

a Determined by expert consensus.

## Discussion

Trachoma, lymphatic filariasis, schistosomiasis, onchocerciasis, and soil-transmitted helminth infections are found in overlapping populations; are controlled through broadly similar, often complementary, strategies involving MDA; and are mapped and monitored by sampling individuals from the population-at-risk using strategies that are also broadly similar but different in detail. Programmes for their control and elimination require improved diagnostic tools to guide decisions on the required intensity, frequency, and duration of intervention and to conduct surveillance for re-emergence of infection after elimination. Similarities between target product profiles (Tables 1 and 2) suggest the feasibility and desirability of integration of diagnostic approaches.

In many areas in which NTDs are highly endemic, basic health infrastructure is sparse or non-existent, and there are few trained personnel. Local laboratories may not have access to refrigeration, reliable power, or piped water; have highly variable capacity for performing diagnostic assays; and the capacity they do have is in general insufficient to meet existing diagnostic requirements of local clinical services. They are therefore ill-equipped to take on the extra burden of generating data to feed into NTD elimination programmes without provision of additional money, staff, training, equipment, reagents, and utilities—or robust technologies that could perform well despite limitations to supply of these resources.

The ideal integrated system might therefore be a portable, self-contained diagnostics platform, capable of performing multiplex assays for several infections of interest on one or a small number of sample types. A system employing microfluidics (“lab-on-a-chip”) [12], [13], [14] technology could fulfil these requirements. The platform should be able to simultaneously undertake multiple roles in different NTD control programmes, each of which might be at various points of evolution within a given population. For example, in a district that had been hyperendemic at baseline for trachoma, soil-transmitted helminths, and lymphatic filariasis but in which interventions had already been in progress for a number of years, the platform would be capable of accurately detecting reductions in ocular *C. trachomatis* infection, whilst simultaneously measuring the prevalence of soil-transmitted helminth infection and monitoring for post-elimination re-emergence of lymphatic filariasis. Since diseases of potential interest will vary from one population to the next, a modular format would provide opportunities to swap diagnostic capacity for particular infections in and out of the platform according to global, regional, or local priority. For example, in onchocerciasis-endemic areas, the capacity to test for loaiasis at the same time as measuring the prevalence of *O. volvulus* infection would benefit programmes [15]. Equally, the platform should be adaptable for the assessment of the community prevalence of HIV infection, malaria parasitaemia or anti-malaria antibody, and/or seroprevalence of antibodies to measles, rubella, or hepatitis B surface antigen following vaccination campaigns.

Our vision can be conceptualised as the delivery of two linked components: a hardware module, on which samples will be processed, and various elements of software, including both the assays themselves and the algorithms to guide their use in the field. To ensure that any new technologies are ready for both registration and end use, field personnel, programme managers, regulatory agencies, ministries of health, and other key stakeholders should be involved in platform development and evaluation.

In addition to the potential savings to existing vertical control programmes that would become possible through integration of diagnostic tools, this approach has several other potential advantages.

First, it makes conducting surveys to rule out specific diseases easier and more cost-effective. This can occasionally yield surprising results. In Burundi in 2007, examination for trachoma was included alongside fieldwork conducted nationally to estimate the prevalences of schistosomiasis and soil-transmitted helminths, in order to confirm the long-held belief that Burundi was trachoma-free. Active trachoma was found in children throughout the country, and trachoma control activities including azithromycin MDA commenced in 2011 in three districts.

Second, proof-of-concept of an integrated diagnostics platform could facilitate programme planning for other infections for which control strategies are in the early stages of development. An October 2009 WHO expert consultation discussed recent work piloting taeniasis elimination in Peru and possible MDA approaches for food-borne trematode infections. These diseases may have global control initiatives developed in the foreseeable future.

Third, establishing capacity for reliable diagnosis of what have hitherto been the most neglected diseases could catalyse a frame-shift in the global health community's vision of developing world laboratory science. A diagnostics platform that could be configured to generate community- or individual-level data for any of the infections already mentioned as well as perform tests for (for example) sexually transmitted infections, human African trypanosomiasis, or leishmaniasis would represent a game-changing advance in the fight against infectious diseases.

World Health Assembly Resolution 60.29 on Health Technologies recognizes that medical devices are indispensable tools for prevention, diagnosis, treatment, and rehabilitation in health care [16]. It is widely accepted that the availability of, and access to, appropriate and affordable health technologies in low- and middle-income countries remain inadequate. In 2010, WHO held the first Global Forum on Medical Devices [17], which featured selected technological innovations that could improve global health. The innovators identified financing, manufacturing partners, and distribution channels as their top three challenges in getting their technologies into resource-limited settings. WHO undertook to continue to interact with industry, funding agencies, academia, and international organizations to raise awareness of the need to design, produce, and commercialize innovative, accessible, and robust technologies which address the needs of health systems particularly in low-resource settings. The development, evaluation, and deployment of an integrated platform to monitor progress towards NTD elimination would be consistent with this WHO vision.

## Supporting Information

Table S1 Performance against the ASSURED criteria [11] of existing diagnostic tools for the neglected tropical diseases employing mass drug administration.
(DOC)
