# Supplementary material for: A Diagnostics Platform for the Integrated Mapping, Monitoring, and Surveillance of Neglected Tropical Diseases: Rationale and Target Product Profiles
Source: PLoS Negl Trop Dis. 2012 Jul 31;6(7):e1746. doi: 10.1371/journal.pntd.0001746 (PMC3409112; doi:10.1371/journal.pntd.0001746)
Supplement: Table S1 — Performance against the ASSURED criteria [11] of existing diagnostic tools for the neglected tropical diseases employing mass drug administration. (DOC) [file pntd.0001746.s001.doc]

Table A: Performance against the ASSURED^1^ criteria of existing diagnostic tools for the NTDs employing MDA

|  | **Lymphatic filariasis (*Wuchereria bancrofti*-endemic areas)** | **Lymphatic filariasis (*Brugia malayi*-endemic areas)** | **Trachoma** | **Schistosomiasis** | **Onchocerciasis (OEPA countries)** | **Onchocerciasis (OCP/APOC countries)** | **Soil-transmitted helminths** |
| --- | --- | --- | --- | --- | --- | --- | --- |
| **Tool in current programme use** | Prevalence of antigen (using the immunochromato-graphic card test or an ELISA-based assay using Og4C3 mononclonal antibody) | Prevalence of BmR1 antibody | Prevalence of the clinical sign “trachomatous inflammation-follicular” (TF) in 1-9 year-old children | Prevalence of eggs in urine or stool, usually examined after specimen concentration^2^ | Prevalence of antibodies directed against *O. volvulus* antigens in school-aged children | Prevalence of subcutaneous nodules using the “Rapid Epidemiological Mapping of Onchocerciasis” method | Prevalence of eggs in stool, usually examined after specimen concentration |
| **Affordability** | USD3-5 per test | USD3-5 per test | ~USD5,000 per district | USD1-2 per test;  ~USD7,000 per district | USD1-2 per test | Dependent on district size, transport costs and per diems | USD1-2 per test |
| **Sensitivity for infection** | Adequate to inform programmatic decisions about stopping MDA | Adequate to inform programmatic decisions about stopping MDA | Adequate to inform programmatic decisions about starting MDA | Low: day-to-day fluctuations of egg excretion make identifying low worm burdens difficult | Test not yet validated for decision making in Africa | Low: nodules may be difficult or impossible to palpate. In areas in which the prevalence of palpable nodules is <20%, only clinic-based treatment is recommended, allowing transmission to persist in these settings, as some infected individuals do not receive treatment | Low: day-to-day fluctuations of egg excretion make identifying low worm burdens difficult |
| **Specificity for infection** | Adequate to inform programmatic decisions about stopping MDA; inadequate for post-elimination surveillance | Adequate to inform programmatic decisions about stopping MDA; performance in the context of post-elimination surveillance unclear | Variable; decreases following MDA and therefore inadequate for monitoring and the stopping decision | High | Test not yet validated for decision making in Africa | Low | High |
| **User-friendliness (Is it simple to perform, requiring minimal training?)** | Yes (ICT). ELISA-based assay requires laboratory-trained personnel. | No: requires laboratory-trained personnel | No: reliable clinical trachoma grading requires training and experience | No: stool often difficult to collect on-demand in the field; parasitological diagnosis requires training and validation, and is labour-intensive and time-consuming | No: requires laboratory-trained personnel | Yes | No: stool often difficult to collect on-demand in the field; parasitological diagnosis requires training and validation, and is labour-intensive and time-consuming |
| **Robustness (Absence of requirement for refrigerated storage)** | No | No | Yes | Yes | No | Yes | Yes |
| **Equipment free?** | ELISA-based assay requires a laboratory | No: requires a laboratory | No: requires binocular loupes | No: requires light microscope | No: requires a laboratory | Yes | No: requires light microscope |
| **Deliverable to those who need it?** | No: current laboratory capacity insufficient to handle centralised processing of specimens at programme scale | No: current laboratory capacity insufficient to handle centralised processing of specimens at programme scale | Yes | Yes | No: assays currently carried out in a limited number of laboratories | Yes | Yes |

APOC= African Program for Onchocerciasis Control, ELISA=enzyme-linked immunosorbent assay, MDA=mass drug administration, NTDs=neglected tropical diseases, OCP=Onchocerciasis Control Program, OEPA= Onchocerciasis Elimination Program in the Americas

1. Mabey et al include two criteria for the “R” of the ASSURED acronym: robustness (absence of a requirement for refrigerated storage; included here) and rapidity (whether or not it enables treatment at first visit). We have excluded the latter criterion for the purposes of evaluating diagnostic tools for community classification, since the rapidity of testing in this context is relatively unimportant.

2. Serology is currently used (in combination with stool microscopy) in China to screen for *S. japonicum* infection.
